# Native *putA* Overexpression in *Synechocystis* sp. PCC 6803 Significantly Enhances Polyhydroxybutyrate Production, Further Augmented by the *adc1* Knockout Under Prolonged Nitrogen Deprivation

**DOI:** 10.3390/ijms26167815

**Published:** 2025-08-13

**Authors:** Suthira Utharn, Peter Lindblad, Saowarath Jantaro

**Affiliations:** 1 Laboratory of Cyanobacterial Biotechnology, Department of Biochemistry, Faculty of Science, Chulalongkorn University, Bangkok 10330, Thailand; 2 Microbial Chemistry, Department of Chemistry—Ångström, Uppsala University, Box 523, SE-75120 Uppsala, Sweden; peter.lindblad@kemi.uu.se

**Keywords:** PHB, *Synechocystis* sp. PCC 6803, proline oxidase, arginine decarboxylase, glutamate

## Abstract

This study highlights a new avenue to improve polyhydroxybutyrate (PHB) productivity by optimizing genes related to arginine catabolism, which influences nitrogen metabolism in cyanobacteria based on the carbon/nitrogen metabolism balance. In the *Synechocystis* sp. PCC 6803 wild type (WT) and its *adc1* mutant (Δ*adc1*), the native *putA* gene, responsible for the oxidation of proline to glutamate, was overexpressed to create the OX*PutA* and OX*PutA*/Δ*adc1* strains, respectively. PHB accumulation was considerably higher in OX*PutA* and OX*PutA*/Δ*adc1* under the nitrogen-deprived condition than in strains that overexpressed the *proC* gene, involved in proline synthesis. The increased transcript level of *glgX*, associated with glycogen degradation, confirmed that glycogen served as the primary carbon source for PHB synthesis under nitrogen stress without any carbon source addition. Furthermore, proline and glutamate level changes helped cells deal with nitrogen stress and considerably improve intracellular carbon/nitrogen metabolism. As indicated by elevated levels of *proA* and *argD* transcripts as well as chlorophyll *a* accumulation, this impact was most noticeable in strains that overexpressed *putA*, which was crucial for the synthesis of glutamate, a precursor for important metabolic pathways that respond to nitrogen stress. Therefore, our metabolic model presents PHB-producing strains as promising candidates for biomaterial biotechnology applications in medical and agricultural fields.

## 1. Introduction

A significant worldwide environmental problem, plastic pollution has drawn much scientific attention in the past ten years, which affects terrestrial, atmospheric, and aquatic ecosystems and poses considerable risks to biodiversity and human health [1,2]. The growing recognition of bioplastics as an acceptable replacement for addressing the sustainability and environmental problems associated with plastics derived from fossil fuels has marked an extensive shift in recent years [3]. Many microorganisms, especially cyanobacteria, produce polyhydroxybutyrate (PHB), a form of bioplastic, as an internal storage polymer [4]. PHB has higher permeability to CO_2_ and O_2_, is highly biodegradable in a variety of environmental conditions, and allows for more processing flexibility [5]. PHB accumulation usually happens when there is an excess of carbon and nutritional stressors, such as inadequate availability of nitrogen or phosphorus [6,7,8,9,10]. The research and application of microalgae will ultimately assist in achieving the Sustainable Development Goals (SDGs) of the UN, which include goals to be applied to food, water purification, renewable energy, environmental management, and the manufacturing of chemicals like biofertilizers, cosmetics, and medical supplies for a circular bio-based economy [11,12].

**Figure 1:**
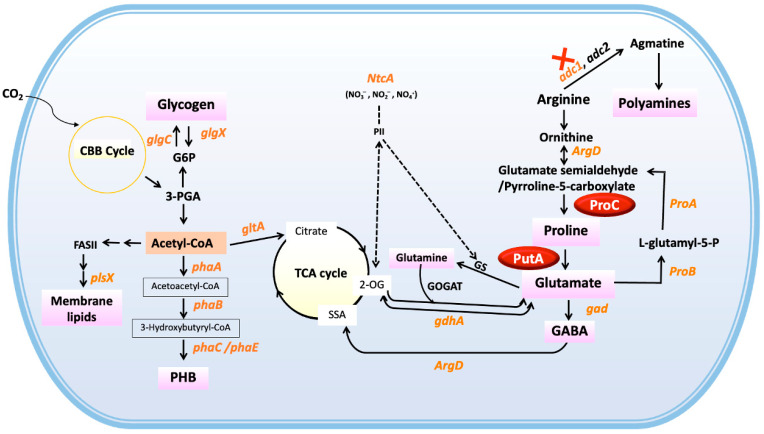
*Synechocystis* sp. PCC 6803: A summary of the polyamine-proline-glutamate pathways connected to the tricarboxylic acid (TCA) cycle, and related glycogen and polyhydroxybutyrate (PHB) biosynthetic processes (modified from [9]). It shows how nitrogen limitation in cyanobacteria increases 2-OG, activating the nitrogen regulator NtcA. NtcA enhances the TCA cycle and promotes glutamate biosynthesis through PutA and ProC catalysis, increasing glutamate and proline levels. Abbreviations for genes and metabolites are shown in the abbreviations section.

In Figure 1, Gram-negative bacteria known as cyanobacteria have all the benefits of photosynthetic microorganisms, most notably the capacity to directly convert carbon dioxide and solar energy into valuable molecules, including lipids, glycogen, and poly-β-hydroxybutyrate (PHB) [9]. For the arginine metabolism, arginine acts as a hub metabolite that can be converted into two pathways: the polyamine biosynthesis and ornithine, which is connected to both the urea cycle and the direction of proline and glutamate production [13,14]. The enzyme arginase catalyzes the initial step of arginine catabolism, converting arginine into ornithine. Ornithine is then processed by the enzyme ornithine transaminase, resulting in the formation of either glutamate semialdehyde or pyrroline-5-carboxylate [13]. It is important to note that the *slr1022* or *ArgD* gene is involved in several metabolic pathways, including the metabolism of arginine and GABA. It has been reported to function as N-acetylornithine aminotransferase, ornithine aminotransferase, and gamma-aminobutyric acid aminotransferase in *Synechocystis* sp. PCC 6803 [9,15]. Next, a key enzyme in the production of proline is Δ^1^pyrroline-5-carboxylate reductase (also known as P5CR or ProC), which uses NADPH or NADH as a cofactor to convert pyrroline-5-carboxylate (P5C) to proline. Proline is subsequently converted to glutamate by the enzyme proline oxidase in proline utilization A (PutA), and three enzymes (ProA, ProB, and ProC) reversibly convert L-glutamate to L-proline concurrently [16]. This capability is rendered possible by glutamate residues that exhibit significant stability in gamma-turn-type geometry. Strong intramolecular (type 2B) H-bond interactions (~1.8 Å) support the backbone N-atom, which is close in proximity to the sidechain C-delta atom, its cyclization partner [17]. Moreover, two different ammonium assimilation routes to generate glutamate include one that is mediated by glutamate dehydrogenase (GDH) and the other that is successively carried out by glutamine synthetase (GS) and glutamate synthase (GOGAT) [18]. The GDH enzyme, which is encoded by *gdhA* in *Synechocystis* sp. PCC 6803, preferentially catalyzes the synthesis of glutamate rather than the reverse process that produces ammonia and 2-oxoglutarate (2-OG) [19]. To maintain C/N homeostasis during nitrogen starvation, cyanobacteria use a sophisticated signal transduction network that senses metabolites like 2-oxoglutarate (2-OG) and cAMP. Increased 2-OG levels under nitrogen depletion enhance NtcA’s binding affinity to DNA and its ability to regulate gene expression involving in nitrogen and carbon uptake, assimilation, and storage [20,21]. During chlorosis induced by nitrogen stress, *Synechocystis* not only disrupts its photosynthetic machinery but also accumulates substantial amounts of biopolymers, such as glycogen and polyhydroxybutyrate (PHB) [9]. Following the onset of nitrogen starvation, glycogen synthesis serves as the primary short-term sink for freshly fixed CO_2_. Additionally, a significant portion of the carbon in PHB originates from internal carbon sources, like glycogen, derived from photosynthetically produced cyanobacteria under nitrogen-starved conditions, rather than from freshly fixed CO_2_ [22,23]. On the other hand, β-ketothiolase (*phaA*), acetoacetyl-CoA reductase (*phaB*), and heterodimeric PHB synthase (*phaE* and *phaC*) catalyze an acetyl-CoA precursor, which is the starting point for the PHB synthesis pathway found in *Synechocystis* sp. PCC 6803 [7,24,25]. Remarkably, it was previously demonstrated that a transposon-randomly modified *Synechocystis* sp. PCC 6803 with a lack of the *proA* gene encoding gamma-glutamyl phosphate reductase had a higher accumulation of PHB [26]. It has been established that *proC* overexpression in *Synechocystis* sp. PCC6803 with *adc1* disruption, leading to lower polyamine production, increases PHB storage by obtaining more acetyl-CoA flow, especially in the presence of nitrogen and phosphate deficiency [9].

In this work, we overexpressed the native *putA* gene encoding proline oxidase (proline utilization A, PutA) in *Synechocystis* sp. PCC6803 wild-type and *adc1* mutant settings to increase the glutamate synthesis necessary for the TCA cycle and other biomolecule syntheses. Applying the nitrogen deficit to all strains, PHB accumulation was significantly elevated in both the *putA*-overexpressing strain (OX*PutA*) and OX*PutA* with *adc1* knockout (OX*PutA*/Δ*adc1*), in particular at day 7 of treatment.

## 2. Results

### 2.1. Overexpression of Native putA Gene in Synechocystis sp. PCC 6803

**Figure 2:**
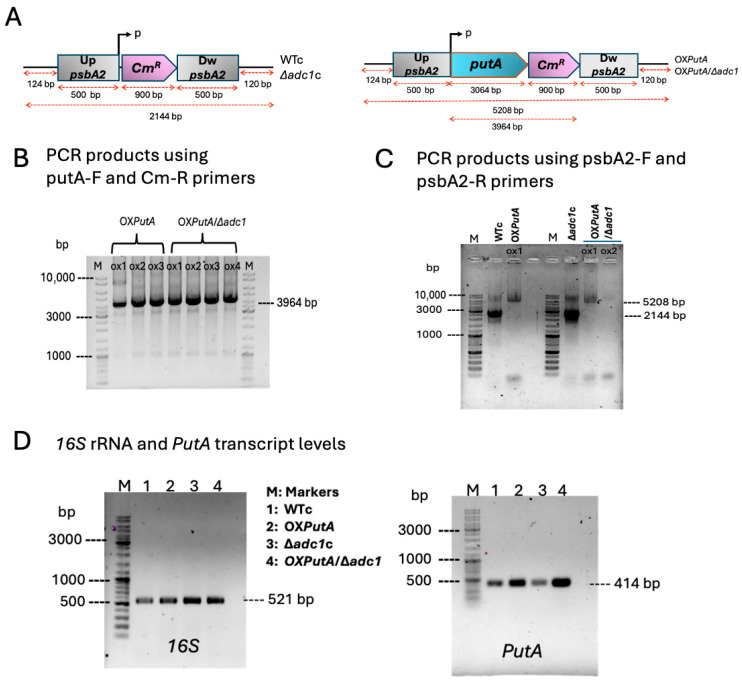
Genomic maps (**A**–**C**) and transcript levels (**D**) of *Synechocystis* sp. PCC 6803 strains. (**A**) The *Cm^R^* gene fragment was recombined between the conserved *psbA2* gene regions in the wild-type control (WTc) and *adc1*-deficient mutant control (Δ*adc1*c) genomes of *Synechocystis* sp. PCC 6803. Similarly, OX*PutA* and OX*PutA*/Δ*adc1* strains were constructed by double homologous recombination using the *putA*-*Cm^R^* fragment, which was found between the *psbA2* gene regions in WT and the Δ*adc1* mutant, respectively. (**B**) For PCR products using putA-F and Cm-R primers, Lane M: GeneRuler DNA ladder, for OX*PutA* strain; Lanes oxs 1–3: three clones no. 1–3, and OX*PutA*/Δ*adc1* strain; Lanes oxs 1–4: four clones no. 1–4 containing a 4.0 kb fragment. (**C**) For PCR products using psbA2-F and psbA2-R primers, Lane M: GeneRuler DNA ladder, for OX*PutA* strain; Lanes WTc: negative control of a 2.1 kb fragment of Up*psbA2*-*Cm^R^*-Dw*psbA2*. For OX*PutA*/Δ*adc1* strain, Lane M: GeneRuler DNA ladder, Lane Δ*adc1*c: negative control of a 2.1 kb fragment of Up*psbA2*-*Cm^R^*-Dw*psbA2*, Lanes oxs 1–2: clones no. 1–2, respectively, containing a 5.2 kb fragment of Up*psbA2*-*putA*-*Cm^R^-*Dw*psbA2*. (**D**) Transcript levels of *putA* gene determined by RT-PCR using RT-putA-F and RT-putA-R primers (Supplementary information Table S2) in WTc, Δ*adc1*c, OX*PutA*, and OX*PutA*/Δ*adc1*. The 1% agarose gel electrophoresis of PCR products was performed from cells grown for 7 days in normal BG_11_ medium. The *16S* rRNA was used as reference.

By overexpressing the *putA* gene in both the wild type (WT) and ∆*adc1* mutant strains of *Synechocystis* sp. PCC6803, we first created OX*PutA* and OX*PutA*/∆*adc1* strains by double recombination (Table 1, Figure 2). In our previous work, the *psbA2* gene in the genomes of *Synechocystis* sp. PCC 6803 WT and ∆*adc1* mutant was substituted with a *Cm^R^* cassette to form the WT control (WTc) and Δ*adc1* control (∆*adc1*c) strains, respectively (Figure 2A) [9]. A 3.1 kb native *putA* (or *sll1561*) gene fragment was ligated between the upstream region of the *Cm^R^* cassette and the flanking areas of the *psbA2* gene of the pEERM vector to produce the recombinant plasmid pEERM_putA (Table 1). After transformation, the obtained engineered strains were verified by PCR using the specific pair of primers (Supplementary Information Table S1) to confirm the complete segregation and correct location. To confirm the gene location, PCR products with putA-F and Cm-R primers revealed the correct size of 4.0 kb in OX*PutA* and OX*PutA*/Δ*adc1* strains (Figure 2B). Furthermore, a 5.2 kb Up*psbA2*-*putA*-*Cm^R^*-Dw*psbA2* fragment was fully segregated in OX*PutA* and OX*PutA*/Δ*adc1* strains, according to PCR results using psbA2-F and psbA2-R primers, whereas a 2.1 kb Up*psbA2*-*Cm^R^*-Dw*psbA2* fragment was seen in the WTc and Δ*adc1*c strains (Figure 2C). Nevertheless, RT-PCR using *putA* and *16S* rRNA reference primers revealed that the *putA* gene transcript was clearly overexpressed in the OX*PutA* and OX*PutA*/Δ*adc1* when compared to the WTc and Δ*adc1*c strains (Figure 2D).

### 2.2. Cell Growth Under Normal Growth Condition and Contents of Intracellular Pigments and Other Metabolite Productions

**Figure 3:**
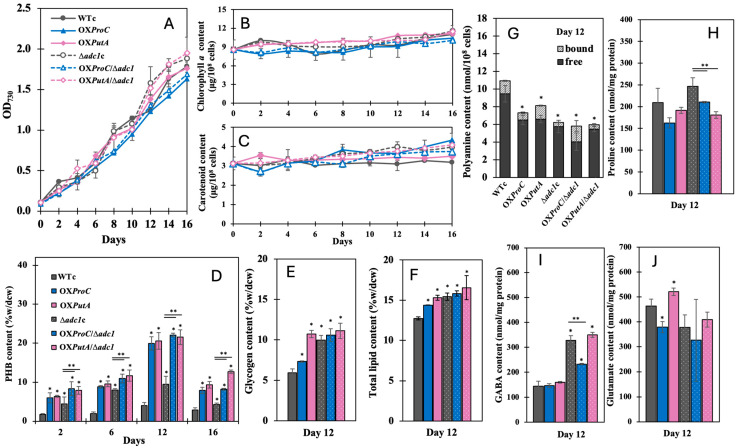
Growth curve (**A**), chlorophyll *a* content (**B**), carotenoid content (**C**), and contents of PHB (**D**), glycogen (**E**), total lipids (**F**), total polyamines (**G**), proline (**H**), GABA (**I**), and glutamate (**J**) of WTc, Δ*adc1*c, OX*ProC*, OX*PutA*, OX*ProC*/Δ*adc1* and OX*PutA*/Δ*adc1* strains. In (**A**–**D**), cells grown in BG_11_ medium for 16 days. In (**E**–**J**), cells were grown in normal BG_11_ medium for 12 days and harvested for metabolite contents. The error bars represent standard deviations of means (mean ± S.D., *n* = 3). In (**D**–**I**), the statistical difference in the results between those values of WTc and that engineered strain is indicated by an asterisk at * *p* < 0.05, and the statistical difference in the results between those values of Δ*adc1*c and that engineered strain with *adc1* disruption is indicated by an asterisk at ** *p* < 0.05.

All strains showed comparable levels of cell growth, with the exception of OX*ProC* and OX*ProC*/Δ*adc1*, which showed more gradual growth than the others (Figure 3A). During the first two days of culture, OX*ProC* and OX*ProC*/Δ*adc1* showed lower amounts of chlorophyll *a* and carotenoids, which subsequently adjusted to levels equivalent to those of other strains in later days (Figure 3B,C). Under the normal BG_11_ condition, PHB contents were increased at day 12 of cultivation, a late-log phase of growth, in particular OX*ProC*, OX*PutA*, OX*ProC*/Δ*adc1*, and OX*PutA*/Δ*adc1*, by about 20% of dry cell weight (dcw) (Figure 3D).

We determined the amount of glycogen that had accumulated based on the maximum PHB level on day 12 of culture under the typical BG_11_ condition. It was discovered that all strains, especially OX*PutA* and those with *adc1* disruption, produced more glycogen than the WTc strain (Figure 3E). Similarly, modified strains had larger levels of total lipids than WTc, which was higher than their glycogen content (Figure 3E,F). Notably, intracellular lipids were proactively accumulated by the WTc and Δ*adc1*c strains at a greater level than glycogen and PHB (Figure 3D–F). Nonetheless, when we looked at arginine catabolism, we discovered that all modified strains showed lower levels of total polyamines than the WTc, especially those with *adc1* disruption (Figure 3G). Furthermore, it appeared that all strains accumulated glutamate at a greater level than proline and gamma-aminobutyric acid (GABA), except for strains with *adc1* disruption (Δ*adc1*c, OX*ProC*/Δ*adc1*, and OX*PutA*/Δ*adc1*), which had a similar level to those of glutamate and GABA under the normal BG_11_ condition at day 12 of culture (Figure 3H–J).

### 2.3. Cell Growth Under the Adaptation Phase Nitrogen-Deprived Condition and Contents of Intracellular Pigments and Other Metabolite Productions

**Figure 4:**
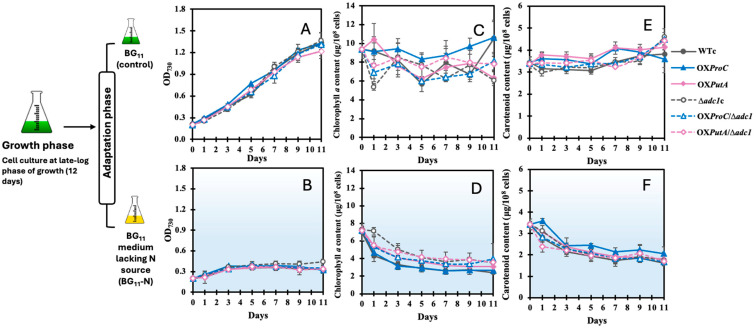
Growth curves (**A**,**B**) and contents of chlorophyll *a* (**C**,**D**) and carotenoids (**E**,**F**) of *Synechocystis* WTc, Δ*adc1*c, OX*ProC*, OX*PutA*, OX*ProC*/Δ*adc1* and OX*PutA*/Δ*adc1* strains adapted in normal BG_11_ medium (**A**,**C**,**E**), and BG_11_ medium with N deprivation (BG_11_-N) (**B**,**D**,**F**) for 11 days. The error bars represent standard deviations of means (mean ± S.D., *n* = 3).

When cells were cultured in BG_11_ medium without a nitrogen source (BG_11_-N) for 11 days, they experienced nitrogen stress during this period, which corresponded to the adaptation phase in our study (Figure 4). Under the normal BG_11_ condition, cells in the late logarithmic growth phase (12 days) were selected due to their high accumulation of PHB (Figure 3D). The cells that were subjected to nitrogen stress appeared to grow less than those in the BG_11_ condition (Figure 4A,B). Despite the fact that cells could not proliferate in the absence of a nitrogen supply, cell growth, as shown by the optical density at 730 nm, remained consistent throughout the course of 11 days. Additionally, it was discovered that intracellular pigments, including chlorophyll *a* and carotenoids, appeared to drop during the first 2 days of the adaptation period before beginning to plateau after 3 days (Figure 4C–F). It is interesting to notice that the OX*ProC* strain contained the most carotenoids and the least amount of chlorophyll *a* of all the strains (Figure 4D,F).

**Figure 5:**
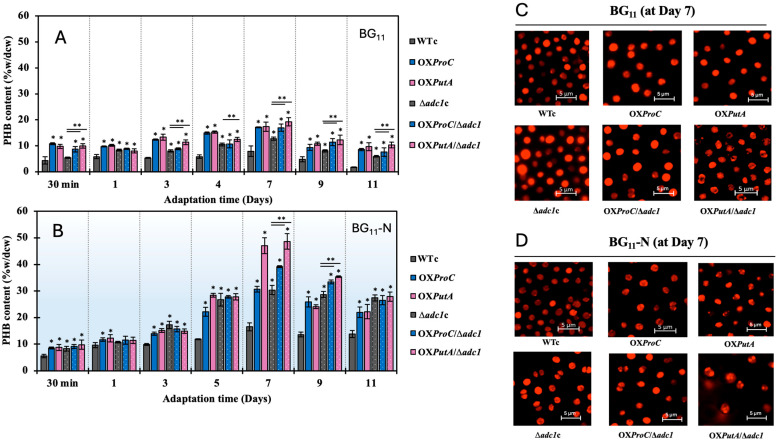
PHB contents (**A**,**B**) and Nile red-stained PHB granules (**C**,**D**) of *Synechocystis* WTc, Δ*adc1*c, OX*ProC*, OX*PutA*, OX*ProC*/Δ*adc1* and OX*PutA*/Δ*adc1* strains. Cells were adapted in normal BG_11_ medium (**A**), and BG_11_ medium with N deprivation (BG_11_-N) (**B**) for 11 days. The error bars represent standard deviations of means (mean ± S.D., *n* = 3). Asterisks (* and ** at *p* < 0.05) denote the statistical difference in results between those WTc values and that engineered strain at each day, and in results between those Δ*adc1*c values and that engineered strain with *adc1* disruption at each day, respectively. In (**C**,**D**), the Nile red-stained PHB granules were performed by using 7 days-adapted cells under normal BG_11_ and BG_11_-N conditions.

**Figure 6:**
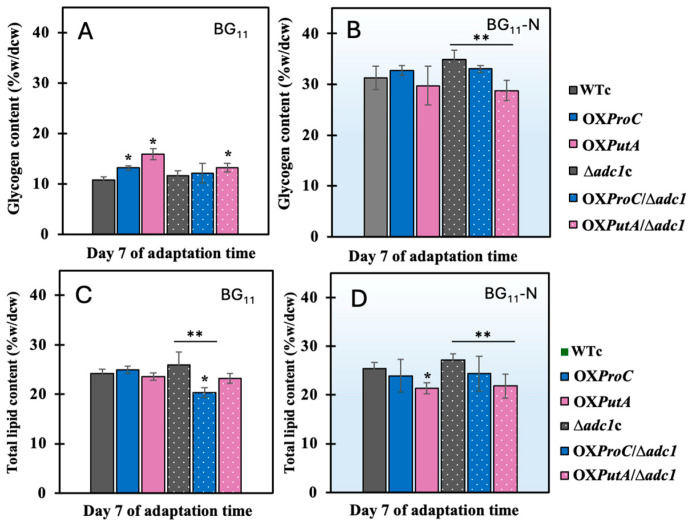
Contents of glycogen (**A**,**B**) and total lipids (**C**,**D**) of *Synechocystis* WTc, Δ*adc1*c, OX*ProC*, OX*PutA*, OX*ProC*/Δ*adc1* and OX*PutA*/Δ*adc1* strains after adapting cells under normal BG_11_ and BG_11_-N conditions for 7 days. The statistical difference in the results between those values of WTc and that engineered strain is indicated by an asterisk at * *p* < 0.05, and the statistical difference in the results between those values of Δ*adc1*c and that engineered strain with *adc1* disruption is indicated by an asterisk at ** *p* < 0.05.

In both the BG_11_ control and BG_11_-N conditions, PHB accumulation during the adaptation period was highest on day 7, particularly in OX*PutA* and OX*PutA*/Δ*adc1*, resulting in 47.1% and 48.6% of dry cell weight, respectively (Figure 5A,B). Cells of all strains under nitrogen stress displayed a greater number of Nile red-stained PHB granules than those under BG_11_ control, which was consistent with the high PHB content (Figure 5C,D). As compared to the BG_11_ control, glycogen, another carbon store, rose as anticipated under nitrogen stress. However, on day 7 of the adaptation period, no significant difference was seen between WTc and any modified strain (Figure 6A,B). It was curious to note that OX*PutA*/Δ*adc1* had a lower level of glycogen under the BG_11_-N condition than the Δ*adc1*c strain, whereas OX*ProC*/Δ*adc1* had a similar level (Figure 6B). Furthermore, by day 7 of the adaptation phase in the BG_11_-N medium, the total lipid content of all strains was completely unaffected when compared to those under BG_11_ control (Figure 6C,D).

**Figure 7:**
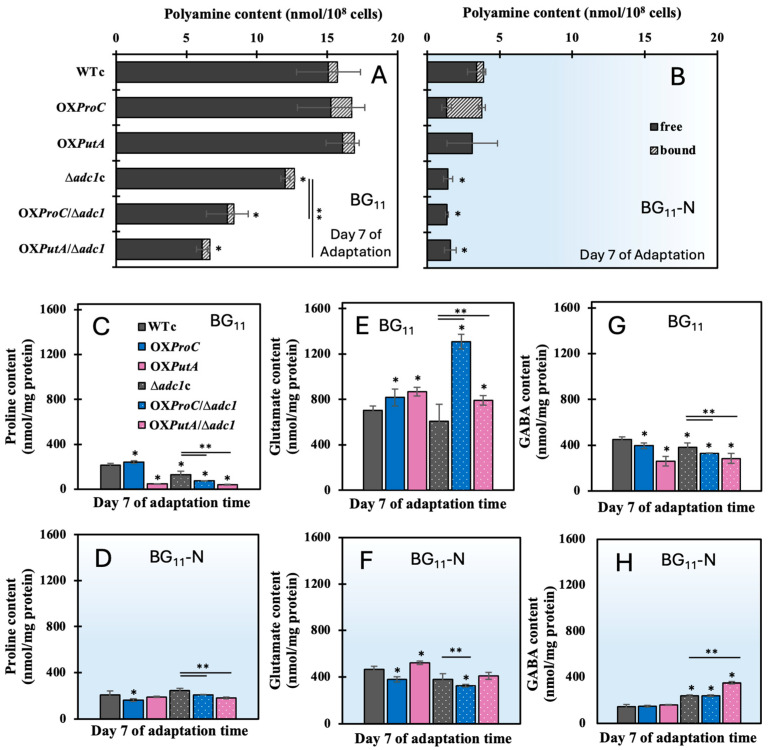
Contents of total polyamines (**A**,**B**), and amino acids including proline (**C**,**D**), glutamate (**E**,**F**), and GABA (**G**,**H**) of *Synechocystis* WTc, Δ*adc1*c, OX*ProC*, OX*PutA*, OX*ProC*/Δ*adc1*, and OX*PutA*/Δ*adc1* strains after adapting cells under normal BG_11_ and BG_11_-N conditions for 7 days. The statistical difference in the results between those values of WTc and that engineered strain is indicated by an asterisk at * *p* < 0.05, and the statistical difference in the results between those values of Δ*adc1*c and that engineered strain with *adc1* disruption is indicated by an asterisk at ** *p* < 0.05.

On the other hand, in the arginine catabolism direction, the decreased content of total polyamines in all strains was induced by the nitrogen deprivation in comparison with those under the BG_11_ condition (Figure 7A,B). On day 7 of the adaptation phase, however, a decrease in proline levels was noted, despite the fact that the total polyamines decreased in the Δ*adc1* mutant strains under the normal BG_11_ condition, resulting in more arginine being converted to ornithine (Figure 7C). Only the OX*ProC* strain contained a higher proline level than the WTc. It is interesting to note that, in contrast to strains under BG_11_ control, OX*PutA* and all Δ*adc1* mutant strains exhibited increased proline accumulation under the BG_11_-N condition (Figure 7D). On the other hand, after day 7 of treatment in BG_11_ medium, the glutamate level in all OX strains was much greater than in WTc and Δ*adc1*c strains (Figure 7E). Comparing all strains to those under the BG_11_ condition, it appears that the nitrogen stress reduced the glutamate accumulation (Figure 7F). Although GABA levels in all engineered strains were lower than in the WTc, it is noteworthy that all *adc1* mutant strains exhibited greater GABA accumulation than the WTc, OX*ProC*, and OX*PutA* strains, particularly OX*PutA*/Δ*adc1* (Figure 7G,H).

**Figure 8:**
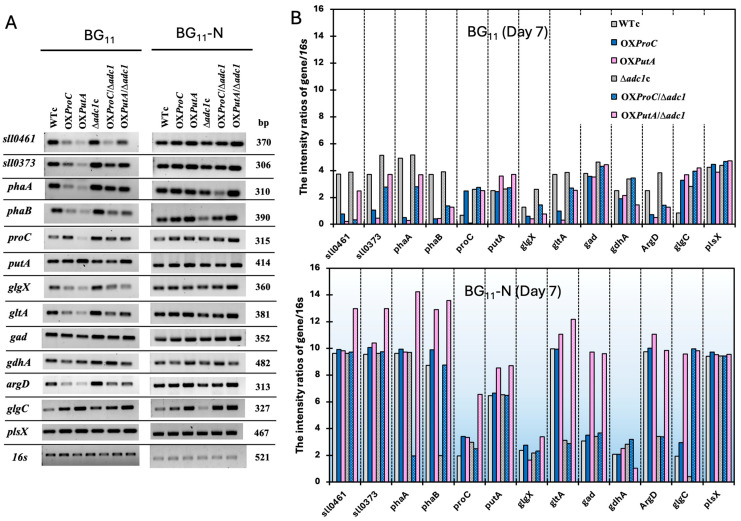
Relative transcript levels (**A**) and relative band intensity ratios (**B**) of *proA* (*sll0461* and *sll0373*), *phaA*, *phaB*, *proC*, *putA*, *glgX*, *gltA*, *gad*, *gdhA*, *argD, glgC*, and *plsX* determined by RT-PCR in *Synechocystis* WTc, Δ*adc1*c, OX*ProC*, OX*PutA*, OX*ProC*/Δ*adc1*, and OX*PutA*/Δ*adc1* strains after adapting in normal BG_11_ and BG_11_-N media for 7 days. The *16s* rRNA was used as a reference control. The relative band intensity ratio was calculated by dividing the band intensity amount of gene transcript by the band intensity amount of *16s* rRNA transcript. All cropped gels were taken from the original images of RT-PCR products on agarose gels as shown in Supplementary Information Figures S1 and S2.

**Figure 9:**
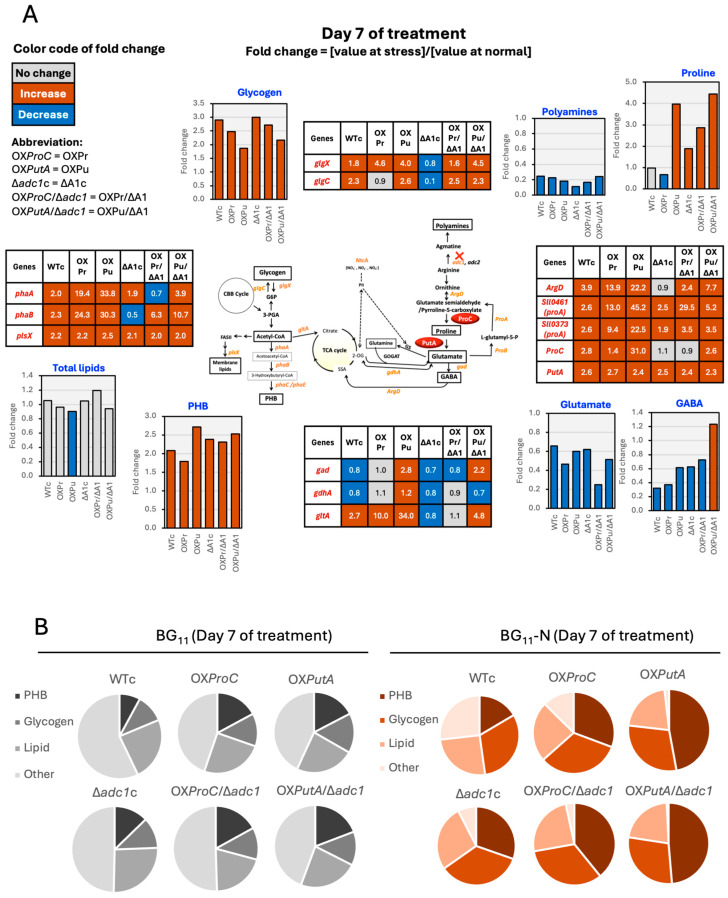
Fold changes in products and relative band intensity ratios (**A**) and proportional distribution of major carbon storage compounds including PHB, glycogen, and total lipids (**B**) in *Synechocystis* WTc, Δ*adc1*c, OX*ProC*, OX*PutA*, OX*ProC*/Δ*adc1*, and OX*PutA*/Δ*adc1* after adapting cells in normal BG_11_ and BG_11_-N media for 7 days. In each table and bar graph, the number and bar represent the fold change of each strain by dividing the value under N stress by the value under normal condition. In A, in terms of box and bar graph coloring, a considerable decrease is denoted by blue, a significant increase by dark orange, and an insignificant change by grey. The data’s statistical differences are at *p* < 0.05. The final yields and the metabolic flux calculation are addressed in the Supplementary Information Table S3.

On the other hand, *proC*-overexpressing strains (OX*ProC* and OX*ProC*/Δ*adc1*) and *putA*-overexpressing strains (OX*PutA* and OX*PutA*/Δ*adc1*) showed greater transcript levels of the *proC* and *putA* genes, respectively, than the WTc, confirming the native gene overexpression (Figure 8A,B). The *argD* gene, which is involved in the reversible conversion of N-acetylornithine to N-acetylglutamate-5-semialdehyde, had the greatest transcript level in the Δ*adc1*c strain under the BG_11_ condition when compared to other strains (Figure 8). Nitrogen deprivation significantly increased the *argD* transcript in all strains by about a 2- to 22-fold increase (Figure 9A) in comparison with those under the normal BG_11_ control, except for the Δ*adc1*c strain, which showed a comparable transcript at day 7. Notably, under the BG_11_ condition, all OX strains showed downregulated transcript levels of *proA* genes, including *sll0461* and *sll0373*, compared to the WTc and Δ*adc1*c strains (Figure 8). In all strains, nitrogen deficiency significantly raised the transcript levels of both *proA* genes (*sll0461* and *sll0373*), which are largely in charge of the elevated glutamate-to-proline conversion (Figure 8). This increase ranged from 1.9 to 45 times the *proA* transcript levels when compared to normal BG_11_ control (Figure 9A).

In addition, *gad* transcript levels for genes involved in GABA production were substantially comparable across strains; however, *putA*-overexpressing strains (OX*PutA* and OX*PutA*/Δ*adc1*) exhibited a modest increase in *gad* transcript levels following nitrogen deprivation (Figure 8) with 2.8- and 2.2-fold increases, respectively, compared to those under normal BG_11_ control (Figure 9A). For the reversible conversion of 2-OG and ammonia to glutamate, under the BG_11_ condition, the transcript level of the *gdhA* gene was upregulated in Δ*adc1*c and OX*ProC*/Δ*adc1* in comparison with the WTc (Figure 8). Curiously, the nitrogen-deprived condition did not induce the increased *gdhA* transcript level when compared to the normal BG_11_ control (Figure 8), except for the OX*PutA* strain, which showed a small increase of about 1.2-fold (Figure 9A). In the conversion of acetyl-CoA to citrate in the TCA cycle, the high transcript amount of *gltA* under the BG_11_ condition was displayed in the Δ*adc1* mutant strains, especially the Δ*adc1*c strain (Figure 8). Striking upregulation of the *gltA* transcript level was induced by the BG_11_-N condition in WTc, OX*ProC*, OX*PutA*, and OX*PutA*/Δ*adc1*, with 2.7-, 10-, 34-, and 4.8-fold increases, respectively (Figure 9A).

On the other hand, in all modified strains, the levels of the *glgC* transcript were higher compared to the WTc under the normal BG_11_ condition, indicating increased glycogen synthesis. Furthermore, in the Δ*adc1*c strain, the *glgX* gene transcript exhibited higher levels than those observed in the WTc, suggesting enhanced glycogen breakdown (Figure 8). However, all strains except the Δ*adc1*c showed higher transcript levels of the *glgX* gene for glycogen breakdown under the BG_11_-N condition than under BG_11_ control, ranging from a 1.6- to 4.6-fold increase (Figure 9A). In addition, it is important to note that the Δ*adc1*c also contained a lower level of the *glgC* transcript for glycogen synthesis under the BG_11_-N condition (Figure 8). For acetyl-CoA, which is a precursor for both lipid and PHB syntheses, the expression of the *plsX* gene for lipid synthesis showed comparable transcript levels across all strains and was also found in higher amounts under nitrogen-limiting conditions (Figure 8). For the PHB synthesis, the transcript levels of the *phaA* and *phaB* genes under the normal BG_11_ control were decreased in OX*ProC* and OX*PutA* strains. The nitrogen deprivation dramatically induced the increased transcript levels of *pha* genes in all strains (Figure 8). Interestingly, it was found that in the OX*ProC*/Δ*adc1* and Δ*adc1*c strains, the transcript levels of *phaA* and *phaB* compensated for each other under the nitrogen-deficient condition for 7 days (Figure 8 and Figure 9A).

## 3. Discussion

Our latest research indicated that disrupting the *adc1* gene, which encodes the arginine decarboxylase enzyme in the polyamine synthetic pathway, leads to increased PHB synthesis [8]. Furthermore, we demonstrated that the simultaneous disruption of *adc1* and overexpression of the *proC* gene enhance the metabolic flux toward proline and glutamate production, resulting in a substantial increase in PHB accumulation. This effect was particularly pronounced under conditions of nitrogen and phosphorus deprivation, coupled with the addition of a carbon source [9]. *Synechocystis* sp. PCC 6803 was shown to produce less proline when the *proC* gene was disrupted, while the *putA* mutant, which lacked the enzyme proline oxidase, gathered a significant amount of the proline metabolite without producing any glutamate [13]. In this study, to gain more understanding and knowledge about the relationship between arginine catabolism and PHB accumulation, we additionally overexpressed the native *putA* gene in both *Synechocystis* sp. PCC 6803 wild type (WT) and its *adc1* mutant strain, generating OX*PutA* and OX*PutA*/Δ*adc1* strains, respectively (Table 1). Once all utilizable nitrogen sources have been exhausted, non-diazotrophic cyanobacteria shift their carbon metabolism to glycogen synthesis and eventually transition into a latent condition called chlorosis, where they remain viable until combined nitrogen is once more accessible [20,27,28]. During nitrogen depletion, glycogen served as a metabolic sink in cyanobacteria; when it was absent, high 2-OG and pyruvate metabolites were released into the medium [29]. However, when the nitrogen supply was sufficient, the glycogen-deficient mutant of *Synechococcus elongatus* PCC 7942 exhibited enhanced carbon partitioning into glutamate and extracellular secretion, while a low intracellular level of glutamate was noted [30]. Our finding indicated that nitrogen deprivation increased the expression of the *putA* gene, which codes for proline oxidase, by more than 6 times (Figure 8 and Figure 9A), although glutamate accumulation was less than that of the normal BG_11_ control (Figure 7F and Figure 9A). We speculated that the spiking rise in *proA* transcript levels (*sll0461* and *sll0373*) during the reaction would indicate that glutamate is catabolized to other metabolic pathways more quickly (Figure 9A). Additionally, under a 7-day nitrogen deficiency, the reduced levels of *gdhA* transcript (Figure 8 and Figure 9A), which is involved in the reversible conversion of 2-oxoglutarate (2-OG) and ammonia to glutamate, suggested a decreased efficiency in glutamate production. Remarkably, the GABA content increased solely in the OX*PutA*/Δ*adc1* strain with regard to its enhanced *gad* mRNA level (Figure 9A). Under extreme conditions like salinity and an acidic pH, GABA functions as a signaling molecule in cyanobacteria [31]. On the other hand, cyanobacteria literally accumulate proline in response to nitrogen shortage, which aids in their ability to withstand the stress that ensues. This proline’s metabolism is linked to other nitrogen-related regulatory mechanisms, and it may also be used as a source of nitrogen [9,32,33]. Our findings indicated that nitrogen deprivation effectively promoted the proline content in OX*PutA* and all Δ*adc1* mutant strains (Figure 9A), even though proline content was generated in a lesser quantity than glutamate (Figure 7).

Moreover, during periods of nutritional constraint, glycogen first plays a crucial role in storing carbon as an energy source and biomass [34] and facilitating photomixotrophic transitions. This helps prevent metabolic imbalances that could otherwise lead to the generation of reactive oxygen species and the inhibition of PSII electron transfer [35]. When nitrogen and phosphate shortages start, *Synechocystis* cells store huge amounts of fixed carbon as glycogen granules [9]. *Synechocystis* cultures revealed that PHB grows gradually and slowly when exposed to prolonged nitrogen deprivation, while glycogen breaks down gradually after first accumulating rapidly when the cells are chlorotic [27]. Rather than additional fixed CO_2_, PHB synthesis is directly sourced from glycogen [23]. In this study, we demonstrated that all strains grown in the typical BG_11_ medium during the late-log phase, particularly those overexpressing the *proC* and *putA* genes, accumulated significantly higher levels of PHB (exceeding 20% of dry cell weight) compared to the WTc. This occurred despite the glycogen content remaining at only about 10% of the dry cell weight on day 12 (Figure 3D). Additionally, the higher transcript level of *glgX* (Figure 8 and Figure 9A) indicates that, despite the fact that the nitrogen deficit led to a greater accumulation of glycogen, it was significantly degraded, particularly in strains that overexpressed *proC* and *putA* genes, including OX*ProC,* OX*PutA*, OX*ProC*/Δ*adc1*, and OX*PutA*/Δ*adc1*, with 4.6-, 4.0-, 1.6-, and 4.5-fold increases, respectively. The *glgC* mRNA level, which is involved in glycogen synthesis, appeared to be increased in all modified strains when compared to the WTc in the normal BG_11_ condition and further enhanced by the nitrogen deprivation stress, with the exception of the Δ*adc1*c mutant (Figure 8). At day 7 of the adaptation phase in the BG_11_-N condition, the higher proportion of glycogen over PHB was found in WTc, OX*ProC*, and Δ*adc1*c strains (Figure 9B). Therefore, the overexpression of native *proC* and *putA* genes substantially enhanced the proline-glutamate direction flow. We speculated that the glutamate excessively produced may subsidize the crucial precursor in many metabolic pathways recovered during the nitrogen shortage. According to *Synechocystis* sp. PCC 6803’s chlorophyll synthesis, which was derived from the glutamate precursor [36], OX*PutA* and OX*PutA*/Δ*adc1* had a high chlorophyll *a* concentration following exposure to nitrogen deprivation (Figure 4D). Additionally, the TCA cycle was able to more effectively regulate the ATP metabolism under nitrogen deprivation because of the increased *gltA* transcript level, which encodes citrate synthase, and decreased *gdhA* transcript level, which encodes glutamate dehydrogenase. As demonstrated by the substantial proportion of PHB in OX*PutA*, OX*ProC*/Δ*adc1*, and OX*PutA*/Δ*adc1* (Figure 9B), acetyl-CoA preferentially flowed to generate PHB abundance in line with the elevated *pha* genes (Figure 9A) while preserving the intracellular lipid level. According to our findings, PHB production was influenced by either *proC* or *putA* overexpression alone, but it improved when combined with *adc1* disruption, as seen in OX*ProC*/Δ*adc1* and OX*PutA*/Δ*adc1* at the highest levels by around 47.1 and 48.6% of dry cell weight, respectively, under the nitrogen deficiency stress. Notably, during prolonged nitrogen stress on days 9 and 11, our findings highlighted the significant impact of *adc1* gene disruption either alone or in combination with the overexpression of the *putA* and *proC* genes on enhancing PHB production (Figure 5). On the other hand, the addition of a carbon source, such as acetate, or nutritional deprivation, or both, were previously used as strategies to increase PHB production [9,37]. In general, PHB formation is favored by high carbon-to-nutrient ratios [38]. In order to increase the high carbon source, the genetic optimization was previously chosen to overexpress the native *RuBisCO* and *phaAB* genes in *Synechocystis* sp. PCC 6803, resulting in high PHB accumulation of about 48–51% of dry cell weight, synergistically induced by the nitrogen and phosphorus deprivation [10]. Our findings, however, indicate that, in addition to the previously mentioned strategies, we can optimize genes associated with the syntheses of arginine to proline and glutamate. By combining these optimizations with nutrient-deficient stress, we can enhance carbon storage in *Synechocystis* sp. PCC 6803, increasing both PHB and glycogen levels. This high-yielding cyanobacteria will facilitate the application of advanced production technologies. In addition to our production strategies, cost-effective cultivation and harvesting procedures must be considered due to the large-scale cultivation.

## 4. Materials and Methods

### 4.1. Construction of Native putA Overexpression in Synechocystis sp. PCC6803

The recombinant plasmid pEERM_putA was initially created and naturally transformed into the Δ*adc1* mutant strain (as previously reported by [8]) and the *Synechocystis* sp. PCC 6803 wild-type (WT) strain in order to produce *putA*-overexpressing strains including OXP*utA*/Δ*adc1* and OX*PutA*, respectively. The pEERM_putA construct was initially generated by PCR-amplifying the *putA* gene fragment using the primer pair PutA-F and PutA-R (as listed in Supplementary Information Table S1), followed by ligation into the *Spe*I- and *Pst*I-cloning sites of the pEERM vector [39]. *Synechocystis* cultures (WT or Δ*adc1*) were grown in the BG_11_ medium until they reached an OD_730_ of 0.3 to 0.5, then collected and resuspended in new medium for transformation. After mixing the plasmid DNA with the cells, they were cultured for the whole night at 28–30 °C with constant light (40–50 μmol photons/m^2^/s). The mixture was cultured for two to three weeks after being plated onto BG_11_ agar supplemented with 10 μg/mL chloramphenicol. For certain stable integration, surviving colonies were subsequently streaked onto BG_11_ plates with higher doses of chloramphenicol (20 and 30 μg/mL). In addition, the empty pEERM vector was transformed into *Synechocystis* WT and Δ*adc1* cells, respectively, to create the *Synechocystis* sp. PCC 6803 wild type control (WTc) and Δ*adc1* control (Δ*adc1*c). These cells represented *Synechocystis* WT and Δ*adc1* with the *Cm^R^* cassette gene in their genomes. The correct transformants were validated by PCR using specific pair of primers (Supplementary Information Table S1) to confirm gene location, and segregation.

### 4.2. Strains and Culture Condition

The typical growth medium, BG_11_, was used to culture *Synechocystis* sp. PCC 6803 WTc, OX*ProC*, OX*PutA*, Δ*adc1*c, OX*ProC*/Δ*adc1*, and OX*PutA*/Δ*adc1*. The growing conditions were 27–30 °C with constant white light illumination at 40–50 μmol photons/m^2^/s. In 250 mL flasks, cultures were cultivated on a shaker at 160 rpm, and the initial optical density at 730 nm (OD_730_) was set to about 0.1. Using spectrophotometry, cell growth was monitored by measuring OD_730_. Initially, all strains were pre-cultivated for 16 days in a normal BG_11_ medium. Following that, cells were collected and exposed to the nitrogen-deprived treatment using a nutrient-depleted medium (BG_11_-N): BG_11_ medium without NaNO_3_, with FeSO_4_ used in place of ferric ammonium citrate. For every strain under the nutrient-limiting condition, the initial OD_730_ of the culture was fixed at roughly 0.2.

### 4.3. Determination of Intracellular Pigments

Intracellular pigments were extracted out of the cultivated cells. First, after transferring one milliliter of the culture to a microcentrifuge tube, the cells were pelleted by centrifuging it for 10 min at 5500 rpm (3505× *g*). To extract the intracellular pigments, 1 mL of dimethylformamide (DMF) was added to mix with cell pellets after the supernatant was removed. The mixture was thoroughly vortexed and centrifuged again at the same speed for 10 min. A spectrophotometer was used to measure the absorbance of the resultant pigment extract at 461, 625, and 664 nm. Pigment concentrations were calculated using the equations outlined by [40,41].

### 4.4. Total RNAs Extraction and Reverse Transcription-Polymerase Reaction (RT-PCR)

*Synechocystis* cells were treated with the TRIzol reagent (Invitrogen, Life Technologies Corporation, Carlsbad, CA, USA) to extract total RNAs. TRIzol solution (500 μL) was used to resuspend the cell pellets, and then 100 mg of glass beads were added. The mixture was vortexed for 30 s at maximum speed after being incubated for 5 min at 70 °C. After that, 100 μL of chloroform was added, vortexed for a brief period, and then centrifuged for 5 min at room temperature at 12,000 rpm (14,383× *g*). After being transferred to a fresh tube, the aqueous phase was mixed with an equivalent amount of cold isopropanol and allowed to sit at room temperature for 10 min. After pelleting the RNAs using centrifugation at 12,000 rpm (14,383× *g*) for 5 min at 4 °C, they were cleaned with 1 mL of 75% cold ethanol and centrifuged once more for three minutes under the same settings. The RNA pellet was allowed to air dry before being dissolved in water treated with DEPC, and the supernatant was disposed of. By treating the total RNAs with 2 μL of DNase I and 2 μL of 10× buffer containing MgCl_2_ and then incubating it for 10 min at 37 °C, genomic DNA was eliminated. A 2.5 μL of 50 mM EDTA was added to stop the reaction, and the mixture was then incubated for 10 min at 65 °C. The ReverTra Ace-α-™ kit (TOYOBO Co., Ltd., Osaka, Japan) was used to create complementary DNA (cDNA). The A_260_/A_280_ ratio, which was around 1.8 and indicated acceptable purity, was used to evaluate the purity of the RNA. RNA samples that had been adjusted to equivalent amounts (1 μg RNA = 1 μg cDNA) were used for cDNA synthesis.

The reaction solution for the reverse transcription process was made up of 1 μg of RNA, 4 µL of 5X RT buffer, 2 µL of dNTP combination (10 mM each), 1 µL of RNase inhibitor, 1 µL of random primer, and 1 µL of ReverTra Ace-α-™. Once the reaction was mixed with 20 µL of RNase-free water, it was incubated for 20 min at 42 °C and 5 min at 99 °C. The resulting cDNA was used as a template for PCR amplification. The PCR settings using specific primers (Supplementary Information Table S2) for *16s* rRNA were as follows: first, denaturation at 95 °C for 5 min; then, 11 cycles of denaturation at 95 °C for 30 s, annealing at 55 °C for 30 s, and extension at 72 °C for 35 s, with a final extension at 72 °C for 5 min. For all genes used in this study, PCR was started for 5 min at 95 °C, then it was annealed for 30 s at the temperature and cycle stated in Supplementary Information Table S2, and finally it was extended for 35 s at 72 °C. Agarose gel electrophoresis was used to evaluate the PCR products using 1.0% agarose in 0.5× TAE buffer. Quantification of band intensity was determined by Syngene^®^ Gel Documentation (Syngene, Frederick, MD, USA).

### 4.5. HPLC Analysis of PHB Content and Nile Red Staining

To obtain the cell pellets, a 50 mL aliquot of cultivated *Synechocystis* cells was centrifuged for 10 min at 5500 rpm (3505× *g*). A solution containing 800 μL of 98% (*v*/*v*) sulfuric acid and 100 μL of the internal standard adipic acid (20 mg/mL) was used to hydrolyze the pellets for 60 min. After hydrolysis, a 0.45 μm polypropylene membrane filter was used to filter the samples. High-performance liquid chromatography (HPLC) on a Shimadzu LGE system (Kyoto, Japan) with an Inert Sustain C18 column (3 μm, GL Sciences, Tokyo, Japan) running at 1.0 mL/min was used to measure the amount of polyhydroxybutyrate (PHB). The mobile phase was prepared by combining 30% (*v*/*v*) acetonitrile with 10 mM KH_2_PO_4_ buffer (pH 7.4). Using a UV detector tuned at 210 nm, crotonic acid, a consequence of PHB breakdown, was detected (modified from [26]). The injection volume was set at 10 μL. Using the same procedure as the samples, authentic commercial PHB (Sigma-Aldrich, Inc., St. Louis, MO, USA) was utilized as the standard. A percentage of the dry cell weight (dcw) was used to represent the PHB concentration. The 50 mL cell pellet was dried in a drying oven for a whole night at 60 °C to determine the constant dry cell weight.

From the control and treatment groups, one milliliter of *Synechocystis* cell culture was taken for Nile red staining. The cell pellets that were produced were then reconstituted in 3 μL of Nile red staining solution. Following that, 100 μL of 0.9% normal saline was added, properly mixed, and the samples were left to incubate in the dark for the whole night (modified from [9,26]). A microscope (Carl Zeiss, Oberkochen, Jena, Germany) was used for fluorescent imaging at 1000× magnification in order to view the stained cells and PHB granules.

### 4.6. Extraction and Determination of Glycogen Content

Cell pellets were collected and alkaline hydrolyzed [42,43]. A 400 μL of 30% potassium hydroxide (KOH) was added to those cell pellets and then heated for an hour. The samples were hydrolyzed, then centrifuged for 10 min at 4 °C at 12,000 rpm (14,383× *g*). The supernatant was subsequently transferred to a new tube. Glycogen or other carbohydrates were precipitated by adding 900 μL of cold ethanol and letting the mixture sit at −20 °C for the whole night. After that, the precipitated samples were centrifuged once more for 30 min at 4 °C at 12,000 rpm (14,383× *g*). The resultant pellets were dried at 60 °C until almost dry, and the supernatant was discarded.

The pellets were treated with 400 μL of 10% sulfuric acid (H_2_SO_4_) in order to measure the amount of glycogen present. After adding 800 μL of anthrone reagent, the liquid was brought to a boil for 10 min. A spectrophotometer was used to detect the absorbance at 625 nm after the sample had cooled to room temperature [44]. The standard curve was created using authentic glycogen, and the amounts of glycogen were represented as a percentage of dry cell weight (%w/dcw).

### 4.7. Extraction and Determination of Polyamine Content

After centrifuging *Synechocystis* cell pellets for 10 min at room temperature at 5500 rpm (3505× *g*), they were extracted using 5% cold perchloric acid (HClO_4_) [45]. The samples were centrifuged for 10 min at 12,000 rpm (14,838× *g*) following an hour of incubation in an ice bath. Benzoylation was used to derivatize the resultant supernatant and pellet, which accounted for the free and bound polyamine fractions, respectively. Using 1,6-hexanediamine as an internal standard, polyamine analysis was carried out using high-performance liquid chromatography (HPLC; Shimadzu HPLC LGE System, Kyoto, Japan) [modified from [46]]. For derivatization, 500 μL of the 5% HClO_4_ extract was mixed with 1 mL of 2 M NaOH, and then 10 μL of benzoyl chloride was added. After extensive vortexing, the mixture was allowed to sit at room temperature for 20 min. Two milliliters of saturated NaCl were added to stop the reaction, and two milliliters of cold diethyl ether were used to extract the benzoylated polyamines. After transferring the ether phase to a new tube and drying it off, the residue was redissolved in one milliliter of methanol. Prior to injection, the samples were filtered through a 0.45 μm cellulose acetate filter. The same procedure was used to produce and derivatize standard polyamine solutions. An Inertsil^®^ ODS-3 C18 reverse-phase column (5 μm, 4.6 × 150 mm) was used for the analysis, and a UV-Vis detector was used for detection at 254 nm. The mobile phase was a methanol/water gradient (60–100%) with a flow rate of 0.5 mL/min.

### 4.8. Quantification of Proline, Glutamate and GABA Contents

Amino acids such as proline, glutamate, and GABA were detected by HPLC utilizing derivatives of *o*-phthalaldehyde (OPA) and 9-fluorenylmethyl chloroformate (FMOC) [47,48]. After centrifuging 50 mL of *Synechocystis* cell culture at 5500 rpm (3505× *g*) for 10 min at 4 °C, the cell pellets were collected. After washing and resuspending the resultant cell pellets in 10 mM potassium phosphate-citrate buffer (pH 7.6), they were homogenized using an ultrasonic homogenizer (BANDELIN electronic GmbH & Co., Berlin, Germany). The homogenates were centrifuged, and the resulting supernatant was concentrated using a Centrivap concentrator (Labconco Corporation, Kansas City, MO, USA). After extracting metabolites from the concentrated sample using 600 μL of a water/chloroform/methanol mixture (3:5:12, *v*/*v*/*v*), 300 μL of chloroform and 450 μL of water were added. Centrifugation at 5500 rpm (3505× *g*) for 10 min at 4 °C resulted in the collection of the upper aqueous phase, which was then dried off and reconstituted in 200 μL of 0.1 N HCl. Prior to the measurement of amino acids, the solution was filtered through a 0.45 μm membrane. Reverse-phase high-performance liquid chromatography (HPLC) (Shimadzu HPLC LGE System, Kyoto, Japan) was used to evaluate the intracellular amino acids with a UV-Vis detector after they were derivatized using *o*-phthalaldehyde (OPA) and 9-fluorenylmethyloxycarbonyl (FMOC) reagents. An Agilent Zorbax Eclipse AAA analytical column (4.6 × 150 mm, 3.5 μm) together with a guard column (4.6 × 12.5 mm, 5.0 μm) (Agilent Technologies, Santa Clara, CA, USA) was used for separation. The mobile phase consisted of solvent A (ACN:MeOH:water, 45:45:10, *v*/*v*/*v*) and solvent B (40 mM Na_2_HPO_4_, pH 7.8). The amino acid derivatives of FMOC and OPA were detected at 262 nm and 338 nm, respectively. Sarcosine and norvaline were used as internal standards for OPA- and FMOC-derivatized amino acids. Amino acid concentrations were presented in nmol per milligram of protein.

### 4.9. Lipid Extraction and Determination of Total Lipid Content

To extract lipids, 25 mL of *Synechocystis* cell culture was extracted using centrifugation for 10 min at 5500 rpm (3505× *g*). The resultant cell pellets were suspended in 1 mL of a 2:1, *v*/*v* chloroform/methanol solution, vortexed rapidly for 2 min, and then incubated for 2 h at 55 °C. After adding 500 μL of distilled water, the mixture was vortexed once more for 2 min at maximum speed and allowed to stand at room temperature for 10 min. After centrifuging the samples for 10 min at 12,000 rpm (14,838× *g*), the lower chloroform phase, which contained the lipids, was carefully transferred to a new glass tube. The aqueous upper phase and interphase were re-extracted using 500 μL of chloroform, and centrifugation was performed under the same setting to maximize lipid recovery. The last layer of chloroform was collected and combined with the initial extract. A dried lipid extract was obtained for analysis by evaporating the mixed chloroform fractions in a fume hood at room temperature (modified from [10,49]).

Using the acid-dichromate oxidation technique, the total lipid content was determined [50]. Commercial canola oil that was prepared in the same way served as the standard. The dried lipid extract was dissolved with one mL of strong sulfuric acid (98% H_2_SO_4_), and it was then thoroughly mixed by vortexing. A potassium dichromate (K_2_CrO_7_) solution of 1 mL was then added. After that, the mixture was heated up for 30 min at 100 °C. A spectrophotometer was used to measure the absorbance at 600 nm after the mixture had cooled to room temperature and 1 mL of distilled water had been added. A percentage of dry cell weight (%w/dcw) was used to represent the total lipid content.

### 4.10. Statistical Analysis

Microsoft Excel version 16.85 was used to compare the results of the two experiments. The statistical technique used was the two-paired sample *t*-test. For all statistical analyses, a value of *p* < 0.05 was considered statistically significant, and a risk threshold of *p* = 0.05 was applied.

## 5. Conclusions

Genetically modified strains of *Synechocystis* sp. PCC 6803 (OX*ProC*, OX*PutA*, OX*ProC*/Δ*adc1*, and OX*PutA*/Δ*adc1*), which are involved in the arginine to proline and glutamate processes, showed elevated levels of PHB accumulation in the nutrient-deprived condition. Native strains that overexpressed *putA* and *proC* were primarily responsible for the high PHB production, which increased even more when *adc1* disruption in the polyamine biosynthesis was introduced, especially in OX*ProC*/Δ*adc1* under the normal BG_11_ condition. It is noteworthy that when exposed to prolonged nitrogen deprivation, particularly after a 7-day treatment, the difference in increased PHB accumulation is significantly impacted by the disruption of the *adc1* gene alone or in combination with the overexpression of the *putA* and *proC* genes. This study is crucial for understanding the relationship between nitrogen metabolism modification and carbon source accumulation within cells. An important aspect that requires further investigation is the secretion of amino acids or metabolites outside the cell while adapting to stress conditions and its carbon partitioning.

## Figures and Tables

**Table 1:** Strains and plasmids used in this study.

Name	Relevant Genotype	Reference
Cyanobacterial strains		
WT control	WT, *Cm^R^* integrated at flanking region of *psbA2* gene in *Synechocystis* genome	[9]
OX*ProC*	*proC*, *Cm^R^* integrated at flanking region of *psbA2* gene in *Synechocystis* genome	[9]
OX*PutA*	*putA*, *Cm^R^* integrated at flanking region of *psbA2* gene in *Synechocystis* genome	In this study
Δ*adc1* control	Δ*adc1*, *Cm^R^* integrated at flanking region of *psbA2* gene in *Synechocystis* mutant genome	[9]
OX*ProC*/Δ*adc1*	*proC*, *Cm^R^* integrated at flanking region of *psbA2* gene in *Synechocystis* mutant genome	[9]
OX*PutA*/Δ*adc1*	*putA*, *Cm^R^* integrated at flanking region of *psbA2* gene in *Synechocystis* mutant genome	In this study
Plasmids		
pEERM	P*_psbA2_*-*Cm^R^*; plasmid containing flanking region of *psbA2* gene	[10]
pEERM-proC	P*_psbA2_-proC*-*Cm^R^*; integrated between *Spe*I and *Pst*I sites of pEERM	[9]
pEERM-putA	P*_psbA2_-putA*-*Cm^R^*; integrated between *Spe*I and *Pst*I sites of pEERM	In this study

## Data Availability

The original contributions presented in this study are included in the article.

## Supplementary Materials

The following supporting information can be downloaded at: https://www.mdpi.com/article/10.3390/ijms26167815/s1.

## Abbreviations

*adc*	Arginine decarboxylase
*ArgD*	N-acetylornithine aminotransferase
Car	Carotenoids
Chl *a*	Chlorophyll *a*
CO_2_	Carbon dioxide
dcw	Dry cell weight
DMF	N,N-Dimethylformamide
GABA	Gamma-aminobutyric acid
*gad*	Glutamate decarboxylase
*gdhA*	Glutamate dehydrogenase
*glgC*	ADP-glucose pyrophosphorylase
*glgX*	Glycerol-3-phosphate dehydrogenase
*gltA*	Citrate synthase
GOGAT	Glutamate synthase
G6P	Glucose-6-phosphate
GS	Glutamine synthetase
h	Hour
m	Meter
µg	Microgram
µL	Microliter
mL	Milliliter
mM	Millimolar
nm	Nanometer
*ntcA*	The nitrogen control A (NtcA) regulator
OD	Optical density
2-OG	2-Oxogutarate
OX	Overexpressing strain
PII	The signal transduction protein PII
PCR	Polymerase chain reaction
3-PGA	3-Phosphoglycerate
*phaA*	β-ketothiolase
*phaB*	Acetocetyl-CoA reductase
*phaC*	The heterodimeric PHB synthase
*phaE*	The heterodimeric PHB synthase
PHB	Polyhydroxybutyrate
*plsX*	Fatty acid/phospholipid synthesis protein
*ProA*	Gamma-glutamyl phosphate reductase
*ProB*	Gamma-glutamyl kinase
*ProC*	∆^1^pyrroline-5-carboxylate reductase
*PutA*	Proline oxidase in proline utilization A
rbc	Ribulose-1,5-bisphosphate carboxylase/oxygenase
rpm	Revolutions per minute
s	Second
SSA	Succinic semialdehyde
WT	Wild type
WTc	Wild type control
