# Supplementary material for: Native putA Overexpression in Synechocystis sp. PCC 6803 Significantly Enhances Polyhydroxybutyrate Production, Further Augmented by the adc1 Knockout Under Prolonged Nitrogen Deprivation
Source: Int J Mol Sci. 2025 Aug 13;26(16):7815. doi: 10.3390/ijms26167815 (PMC12386471; doi:10.3390/ijms26167815)

Supplementary Information

Table S1. PCR primers for construction recombinant plasmid

Target gene	Name	Oligo sequences	Amplified fragment length (bp)
*put*A	putA-F	5'-ggACTAGTATCGCCCAGAAGATAAGC C-3'	3064
	putA-R	5'-aaCTGCAGGACCCCGATGACTCAAGAAC-3'	
Cm^R	Cm-F	5'-CTCGAGGCTTGGATTCTCTCAC-3'	900
	Cm-R	5'-CTCGAGGCTTGGATTCTCTCAC-3'	
*psbA*2	psbA2-F	5'-CTTTAGCGTTCCAGTGGATATTTGC-3'	3595
	psbA2-R	5'-TTGTAACGGGCGATCGCCTTGGCAA-3'	

Table S2. PCR primers for RT-PCR

Target gene	Name	Oligo sequences	Amplified fragment length (bp)	Annealing temp. (ºC)	Cycles
*put*A	RT- putA -F<br>RT- putA -F	5'- GTGATTTCCCCCTGGAATTT-3'<br>5'- ACCAAAGGCGGAATACACTG-3'	414	56	25
*pro*C	RT-ProC-F<br>RT-ProC-R	5'- GGCTAAGGCTAAGGCCATCT-3'<br>5'- CGAAAACCCATCTTCTCCAA-3'	315	55	25
*pha*A	RT-phaA-F<br>RT-phaA-R	5'- CATGATGGTTTGACGGACAG-3'<br>5'- GACTACAGTTGCCCGCTGTT-3'	310	56	27
*pha*B	RT-phaB-F<br>RT-phaB-R	5'- ATGCCGGTATCACCAAAGA-3'<br>5'- CAATTTCCTCCGGTTTACCA-3'	390	56	27
*pro*A (sll0373)	RT-0373-F<br>RT-0373-R	5'- GGTGAAGGATGTGGAGCATT-3'<br>5'- TCCTCCCTGGTGGTGAGTAG-3'	306	59	25
*pro*A (sll0461)	RT-0461-F<br>RT-0461-F	5'- ACCTTGGACTTGGTGGTGAG-3'<br>5'- CTGGTAAATCCGCATCCAGT-3'	370	59	25
*Arg*D	RT-ArgD -F<br>RT-ArgD -R	5'-CAAGTTGTGGGGCTACGAAC-3'<br>5'-AATTTCCGCCAAACCACTCC-3'	265	59	27
gad	RT-gad-F<br>RT-gad-R	5'-CAGTGAAGCGGAAAGCCTAC-3'<br>5'- AGAACCAATGGTGGAACAGC -3'	352	60	30
*gdh*A	RT- gdhA -F<br>RT- gdhA -R	5'- GCGTTTAAGTCGGGGTTACA -3'<br>5'-TTTCCCCTAAATCGCAGATG -3'	482	61	30
*glt*A	RT- gltA -F<br>RT- gltA -R	5'- ATGAATGCGTCCACCTTTTC -3'<br>5'- GTCCACATTGGGGTAAATGC-3'	381	52	25
*glg*X	RT- glgX -F<br>RT- glgX -R	5'- GAGCTTCATCGAGGACGGAA- 3'<br>5'- GCCCGAATTGGGGTTGCGGG – 3′	360	64	27
*16*s rRNA	RT-16 -F<br>RT-16s-R	5'-AGTTCTGACGGTACCTGATGA- 3'<br>5'-GTCAAGCCTTGGTAAGGTTCT - 3'	521	55	11
plsX	RT- plsX -F<br>RT- plsX -R	5'- AAGGGGTGGTGGAAATGGAA- 3'<br>5'- AAGTACGTCCCTTCCTTCGG- 3'	467	58	30
glgC	RT- glgC -F<br>RT-glgC-R	5'-ACCAATGCCGACATAACCCTTTCCG-3'<br>5'-ATAGGCTTGCAGATTGTGATCACTGGC-3'	327	59	24

Table S3. The final yields of metabolites and the metabolic flux calculation (Figure 9) representing the ratio of the metabolite content under the BG_{11}-N condition divided by the metabolite content under the BG_{11} condition at day 7 of treatment.

Content	Conditions	WTc	OX*ProC*	OX*PutA*	Δ*adc1*c	OX*ProC*/Δ*adc1*	OX*PutA*/Δ*adc1*
PHB (%w/dcw)	BG_{11}	7.92	17.12	17.36	12.75	16.98	19.21
	BG_{11}-N	16.51	30.69	47.09	30.36	39.17	48.65
	Ratios	2.08	1.79	2.71	2.38	2.31	2.53
Glycogen (%w/dcw)	BG_{11}	10.78	13.22	15.93	11.66	12.18	13.26
	BG_{11}-N	31.27	32.77	29.73	34.91	33.04	28.77
	Ratios	2.90	2.48	1.87	2.99	2.71	2.17
Total lipids (%w/dcw)	BG_{11}	24.15	24.93	23.60	25.95	20.36	23.19
	BG_{11}-N	25.44	23.95	21.33	27.17	24.36	21.85
	Ratios	1.05	0.96	0.90	1.05	1.20	0.94
Proline (nmol/mg protein)	BG_{11}	213.69	241.89	48.25	130.88	73.43	40.65
	BG_{11}-N	209.27	162.60	191.48	246.95	210.28	180.76
	Ratios	0.98	0.67	3.97	1.89	2.86	4.45
Glutamate (nmol/mg protein)	BG_{11}	702.87	816.85	866.70	608.25	1307.84	792.27
	BG_{11}-N	463.63	379.45	521.02	377.93	326.79	409.32
	Ratios	0.66	0.46	0.60	0.62	0.25	0.52
GABA (nmol/mg protein)	BG_{11}	450.04	396.14	260.51	382.45	329.45	283.43
	BG_{11}-N	144.99	146.89	160.05	238.69	238.34	350.05
	Ratios	0.32	0.37	0.61	0.62	0.72	1.24
Polyamines (nmol/10^8 cells)	BG_{11}	15.72	16.73	16.91	12.66	8.37	6.66
	BG_{11}-N	3.87	3.77	3.10	1.43	1.37	1.60
	Ratios	0.25	0.23	0.18	0.11	0.16	0.24

Figure S1. Original images of the RT-PCR products in all strains adapted in BG_{11} medium for 7 days. RT-PCR products were separated on a 1% agarose gel electrophoresis. The analyzed genes include *proA* (*sll0461*, 370 bp), *proA* (*sll0373*, 306 bp), *phaA* (310 bp), *phaB* (390 bp), *proC* (315 bp), *putA* (414 bp), *glgX* (360 bp), *gltA* (381 bp), *gad* (352 bp), *gdhA* (482 bp), and *argD* (313 bp), *plsX* (467 bp), *glgC* (327 bp), *16s* rRNA (521 bp) was used as a reference.

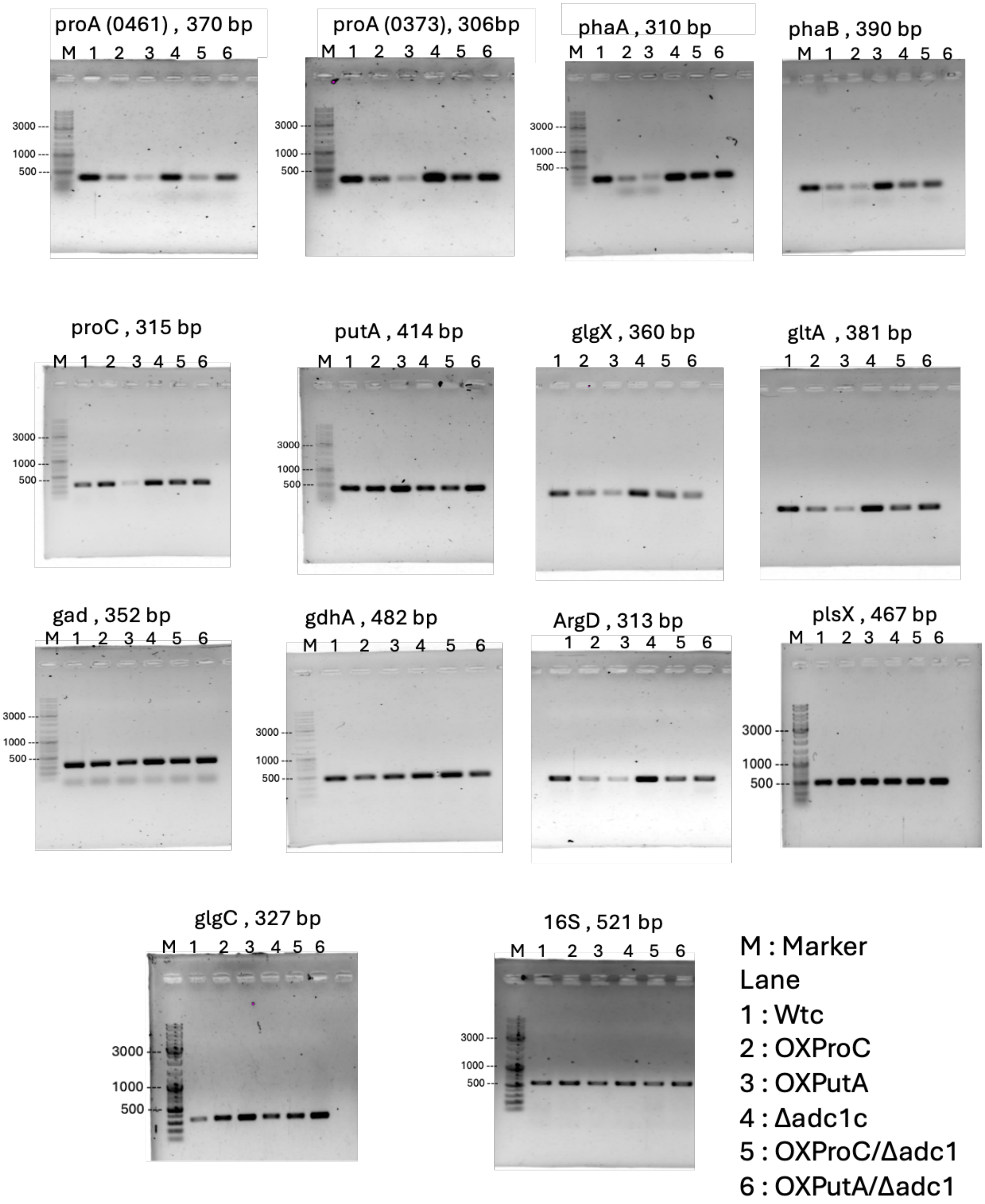

Figure S2. Original images of the RT-PCR products in all strains adapted in BG_{11}-N medium for 7 days. RT-PCR products were separated on a 1% agarose gel electrophoresis. The analyzed genes include *proA* (*sll0461*, 370 bp), *proA* (*sll0373*, 306 bp), *phaA* (310 bp), *phaB* (390 bp), *proC* (315 bp), *putA* (414 bp), *glgX* (360 bp), *gltA* (381 bp), *gad* (352 bp), *gdhA* (482 bp), and *argD* (313 bp), *plsX* (467 bp), *glgC* (327 bp), and *16s* rRNA (521 bp) was used as a reference.

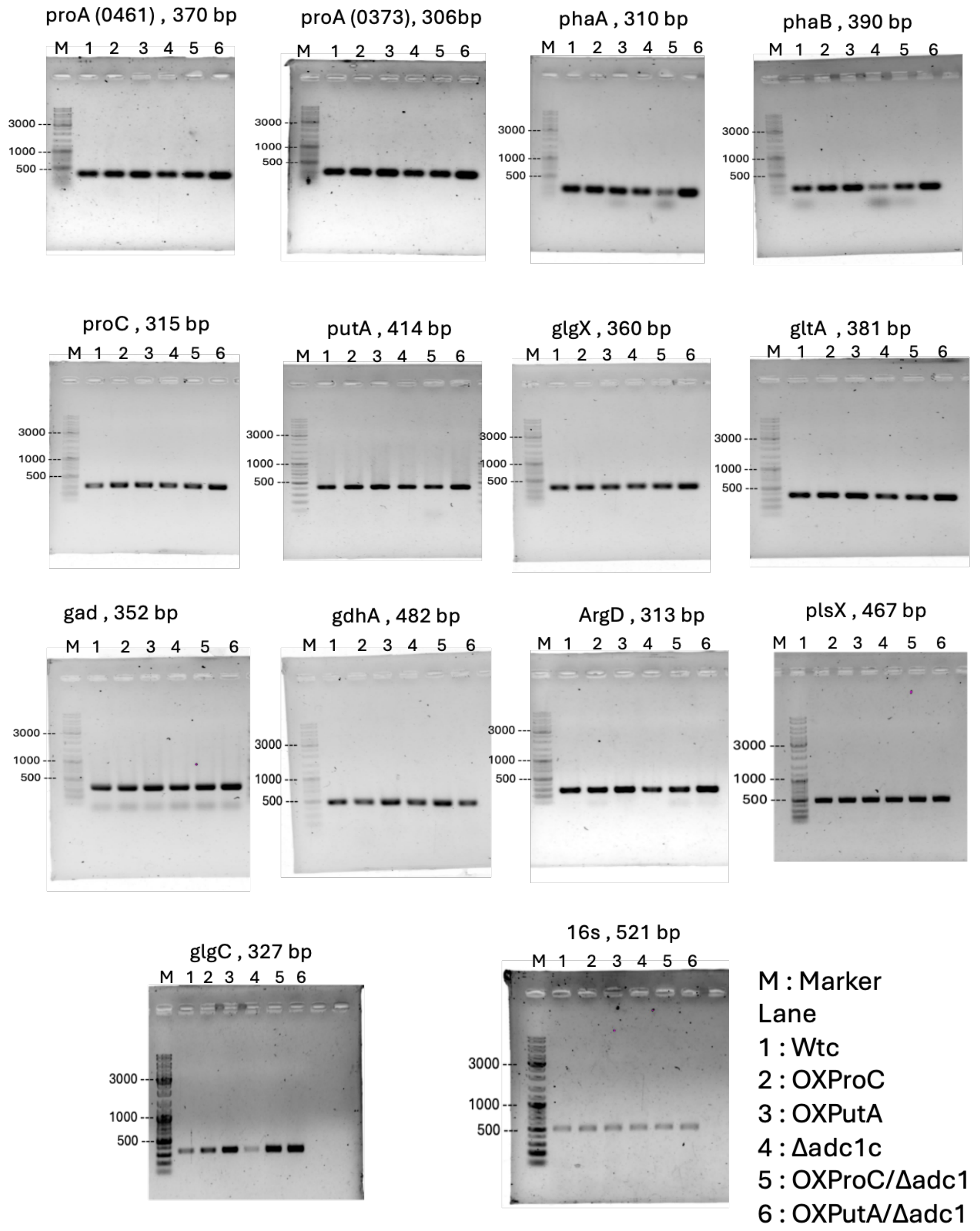

Supplement: Supplementary file 1 [file ijms-26-07815-s001.zip › ijms-3768974-supplementary.pdf]
